# The role of the trithorax group TnaA isoforms in Hox gene expression, and in *Drosophila* late development

**DOI:** 10.1371/journal.pone.0206587

**Published:** 2018-10-29

**Authors:** Marco Rosales-Vega, Adriana Hernández-Becerril, Juan Manuel Murillo-Maldonado, Mario Zurita, Martha Vázquez

**Affiliations:** 1 Departamento de Fisiología Molecular y Genética del Desarrollo, Instituto de Biotecnología, Universidad Nacional Autónoma de México, Cuernavaca, Morelos, México; 2 Departamento de Neurobiología del Desarrollo y Neurofisiología, Instituto de Neurobiología, Universidad Nacional Autónoma de México, Querétaro, Querétaro, México; University of Dayton, UNITED STATES

## Abstract

Regulation of developmental gene expression in eukaryotes involves several levels. One of them is the maintenance of gene expression along the life of the animal once it is started by different triggers early in development. One of the questions in the field is when in developmental time, the animal start to use the different maintenance mechanisms. The trithorax group (TrxG) of genes was first characterized as essential for maintaining homeotic gene expression. The TrxG gene *tonalli* interacts genetically and physically with genes and subunits of the BRAHMA BAP chromatin remodeling complex and encodes TnaA proteins with putative E3 SUMO-ligase activity. In contrast to the phenocritic lethal phase of animals with mutations in other TrxG genes, *tna* mutant individuals die late in development. In this study we determined the requirements of TnaA for survival at pupal and adult stages, in different *tna* mutant genotypes where we corroborate the lack of TnaA proteins, and the presence of adult homeotic loss-of-function phenotypes. We also investigated whether the absence of TnaA in haltere and leg larval imaginal discs affects the presence of the homeotic proteins Ultrabithorax and Sex combs reduced respectively by using some of the characterized genotypes and more finely by generating TnaA defective clones induced at different stages of development. We found that, *tna* is not required for growth or survival of imaginal disc cells and that it is a fine modulator of homeotic gene expression.

## Introduction

Homeotic (Hox) genes determine the segmental identity in *Drosophila*. In *Drosophila* Hox genes are in two complexes, the bithorax (BX-C) and the Antennapedia (ANTP-C) complexes. The initiation of Hox expression in specific segments occurs during embryogenesis and it is controlled by maternal and segmentation genes. Later on the activation or repression are maintained in the appropriate segments by proteins encoded by genes that belong to the trithorax group (TrxG) or the Polycomb group (PcG) respectively. Several TrxG and PcG proteins are involved in chromatin dynamics (reviewed by [1]). *Drosophila* has two types of the SWI/SNF chromatin remodeling complex BRAHMA (BAP and PBAP), which have as a catalytic ATPase, the Brahma protein. These two types have common and specific subunits. Common subunits are Brahma and Moira, while Osa is a specific subunit of BAP. Brahma, Moira and Osa are encoded by TrxG genes [2–4]. *tonalli* (*tna*) is a TrxG gene that was identified because it modifies *brahma* (*brm*), *osa* (*osa*) and *moira* (*mor*) [5].

*tna* encodes TnaA_130_ and TnaA_123_, two TnaA isoforms that presumptively have E3 SUMO ligase activity (see ahead, and [6]). These isoforms are derived either from different transcripts [7] and/or as a result of the processing of some of them [6]. TnaA_130_ and TnaA_123_ isoforms are differentially expressed during development and have specific compartmentalization within the cell [6].

SUMOylation is a post-translational modification similar to ubiquitination that adds a SUMO moiety to target proteins through the action of common activating E1 and conjugation E2 enzymes that in *Drosophila* are represented by single proteins. In contrast, there are several types of E3 ligases that choose or help the SUMOylation of a target protein. SUMOylation of a target protein can change its sub-compartmentalization within the cell or nucleus, can favor a change of partners and/or it can label it for degradation (revised in [8]). The PIAS (Protein Inhibitors of Acivated STAT [Signal Transducers and Activators of Transcription]) family is a subgroup of E3 SUMO ligases that interact physically with E2 enzymes through a canonical 42 amino-acidic residues SP-RING (Siz/PIAS-Really Interesting) zinc finger [9]. TnaA share with the PIAS family the SP-RING [9] but this zinc finger is embedded in a unique 300 amino-acidic residues XSPRING (eXtended SP-RING) domain that is found in a few insect and vertebrate proteins and that is not present in the PIAS proteins [5]. TnaA physically interacts with *Drosophila* SUMO conjugating enzyme E2 *in vivo* and it coimmunoprecipitates with the Osa and Brm proteins from the BRM complex in embryo extracts [6].

Hox gene expression starts early at embryonic stages and prevails late in development. Therefore, it is controlled at each stage and tissue by different selected transcription factors that act on specific regulatory regions of each Hox gene (reviewed in [10]). Thus, it is probable that chromatin accessibility of these regulatory regions is under fine control involving chromatin remodelers and/or modifiers. As a TrxG gene, *tna* is required for the maintenance of expression of Hox genes [5], and adult animals with mutations in *tna* or in *tna* and *brm*, or *osa* mutations, show phenotypes that resemble Hox loss-of-function revealed in adult cuticular structures. One of the characteristics that make *tna* unique among TrxG genes is that it is required late in development [5], being the lethal phase of *tna* third instar larvae and pupal stages [5, 6]. As *tna* was identified as a *brm*-modifier gene and animals with *tna* mutant combinations reach the pharate stage and die before reaching adulthood presenting Hox loss-of-function phenotypes [5], one hypothesis is that its function is required to maintain Hox gene expression by facilitating chromatin remodeling by the BRAHMA BAP complex at these late stages of development. These facts make TnaA protein(s) interesting to study for the function they could have to ensure correct gene expression at these stages of development.

In this work we explored TnaA requirements for the expression of the Hox genes *Ultrabithorax* (*Ubx*) and *Sex combs reduced* (*Scr*), through immunostaining of the respective Hox proteins in imaginal discs of late third instar larvae with mutant *tna* genotypes, or in TnaA defective clones generated at different stages of development. We found that although animals derived from these experiments do present Hox loss-of-function adult cuticular phenotypes, the wild-type domains of Hox expression are not visibly altered in imaginal discs. In contrast, ectopic Hox expression is suppressed in *tna* mutant backgrounds, leading to the conclusion that TnaA finely modulates Hox gene expression in imaginal cells and that its function can only be observed when Hox gene expression is not robustly regulated.

## Material and methods

### Ethics statement

All animals handling was approved by the Instituto de Biotecnología, UNAM, Bioethics Committee, Permit Number 359 (2018/05/04), which follows NOM-062 animal welfare Mexican law. All efforts were made to minimize animal suffering. Animals were sacrificed by CO_2_ euthanasia.

### Fly strains, and genetic procedures

**Fig 1:**
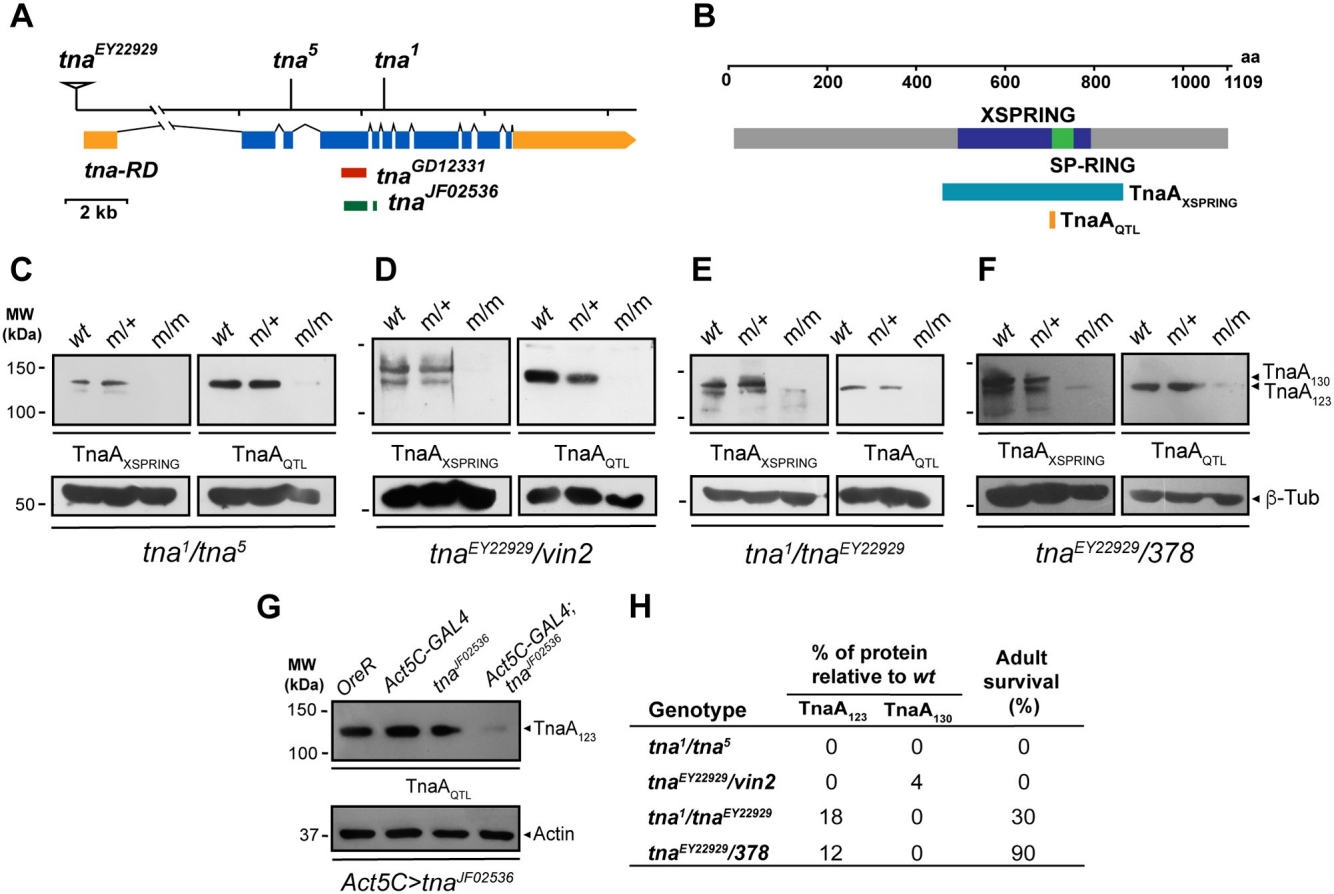
TnaA isoforms present in third instar larvae with different *tna* mutant genotypes. (**A**) *tna* genomic region of the tna-RD transcript ([11], untranslated and translated exons in yellow and blue respectively) indicating the lesions (triangle for insertion and vertical black lines for point mutations) and the RNAi alleles (region targeted, red and green for *tna*^*GD12331*^, and *tna*^*JF02536*^ respectively) used in this work. (**B**) TnaA protein (grey, 1109 residues) indicating the XSPRING (purple) and SP-RING (green). The regions targeted by the polyclonal antibodies are shown, TnaA_XSPRING_ (turquoise) and TnaA_QTL_ (yellow). TnaA isoforms in different third instar larvae with *tna* mutant genotypes. The genotypes are indicated at the bottom of each panel (**C-G**), and they are ordered according to adult survival (**H** and Table 1). The genotypes of the larvae used to prepare the protein extracts tested are *OregonR* (wt), heterozygote *tna*/+ (m/+, Tubby larvae), and heteroallelic *tna* mutant (m/m, non Tubby larvae) (upper part in each panel). Western blots of soluble protein extracts from third instar larvae were probed with TnaA_XSPRING_ (dilution 1:250) and TnaA_QTL_ (1:3000) antibodies as indicated. TnaA_XSPRING_ often detects also a minor 95–110 kDa protein (observed for example in C and D), that it is a *bona fide* TnaA-related product (not seen with preimmune). (**G**) TnaA knockdown in third instar larvae. *tna*^*JF02536*^ RNAi-expression driven by *Act5C-GAL4*. Larvae were raised at 28°C. β-tubulin (C-F), and actin (G) were used as loading controls. Note that survival is observed when TnaA_123_ is present (*tna*^*1*^*/tna*^*EY22929*^, and *tna*^*EY22929*^*/378* genotypes). (**H**) Quantification of TnaA_123_ and TnaA_130_ in larvae with the mutant-indicated genotypes and their adult survival according to Table 1. Note that even low amounts of TnaA_123_ are enough to allow animals to reach adulthood. (*tna*^*1*^*/tna*^*EY22929*^, and *tna*^*EY22929*^*/378* genotypes). The percentage was calculated relative to the amount of each isoform observed in the wild-type *OreR* larvae using the TnaA_XSPRING_ antibody (left in each panel).

The lesions of *tna* alleles and the target of the interference RNAs (RNAi) used in this work are represented in Fig 1A and, unless otherwise noted, they are described in Flybase [7]. Briefly, *tna*^*1*^ and *tna*^*5*^ are EMS-induced mutations. In *tna*^*1*^ Gln 566 changed to a stop codon [5]. *tna*^*5*^ was recovered after EMS mutagenesis in a genetic screen to identify *brm*-interacting mutations. The lesion is a T for A change at base 10,857,881 (genome release version 6) [11] that correspond to the limit between exon 3 and 4 (where exon 1 is UTR) from tna-RD and that affects the splicing of a 451 bp intron present in all *tna* transcripts (J. A. Kennison, personal communication). *tna*^*EY22929*^ is a P{EPgy2} element insertion [12]. *tna* knockdown was achieved by expressing interference RNA (RNAi) from lines *tna*^*GD12331*^ (inserted either in chromosome 2, or in chromosome 3) from Vienna GD collection (vector pGD264, construct ID 12331 Vienna *Drosophila* Resource Center, [13]), and *tna*^*JF02536*^ from Perrimon’s pVALIUM10-derived TRiP [14] collections using different drivers. Drivers used in this work were *Act5C-GAL4* [15] for ubiquitous expression, and *MS1096-GAL4* [16], and *A9-GAL4* [17] were used to drive gene expression to the dorsal region of the haltere pouch. Fly cultures and crosses were performed according to standard procedures. Flies were raised on yeast-molasses media at 25°C unless otherwise noted.

Lethality of individuals carrying heteroallelic combinations of *tna* alleles was determined by counting the Tb^+^ progeny from crosses between parents with *tna* alleles balanced with *In(3LR)TM6B* (*tna*^*+*^) carrying the larval/pupal marker *Tb*^*1*^, and the adult markers *Dr*^*Mio*^, or *Sb*^*1*^. To evaluate pupariation and adult survival rates of heteroallelic *tna* individuals, and the survival of *tna* knocked-down flies, we performed a χ^2^ test (significance set at P<0.05), comparing the number of *tna* heteroallelic animals (*tna/tna*) with the one of their *tna/+* siblings in each genotype. For the eclosion rate analysis, we performed a t-test (P<0.05), comparing the proportion of eclosed/total heteroallelic *tna/tna* pupae (non-Tubby). At least two crosses were performed for each genotype.

Loss-of-function Hox phenotypes were scored in adult animals from *tna*^*1*^/*tna*^*EY22029*^, with *tna* knockdown and from crosses where imaginal *tna*^*1*^ clones were induced (see Induction of Mitotic Clones section), and compared their appearance on control animals derived from crosses without the *tna*^*1*^ *FRT2A* chromosome and thus *tna*^*+*^. Three replicas were performed for each experiment. To evaluate the *Scr* loss-of-function phenotype we scored legs from at least 15 males of each genotype. The percentage was calculated by dividing the number of legs with less than nine teeth per sex comb over the number of total male legs scored. Statistical significance was determined with a t-test (P<0.001).

To analyze the suppression effect of *tna* mutant alleles on ectopic Hox expression in imaginal discs, immunostaining with the respective Hox protein antibody was performed in at least 40 imaginal discs per genotype of interest, derived from at least three independent replicas per genotype. Statistical significance was determined using a t-test (P<0.05) to compare the number of discs from *tna* transheterozygous animals showing ectopic Hox suppression and the number of *Pc*^*3*^ discs showing ectopic Hox expression.

### Antibodies, production and affinity purification of TnaA antibodies

To detect TnaA in this work, we used two polyclonal rabbit antibodies, anti-TnaA_XSPRING_, and anti-TnaA_QTL_ raised against regions of the TnaA_PD_ isoform identified and sequenced by Gutiérrez *et al*., (2003) and reported by Flybase [11]. Rabbit anti-TnaA_XSPRING_ was raised as the one from rat reported in Monribot-Villanueva *et al*., (2013) immunizing animals with a purified GST fusion protein harboring the entire XSPRING domain contained in aminoacids 433–856 of TnaA. Anti-TnaA_QTL_ was raised against the 14-mer QTLHKRNLLPLEHS peptide (aminoacids 691–704) by New England Peptide. Both antibodies (Fig 1B) were affinity-purified from total sera.

For Western blot assays, affinity-purified primary rabbit anti-TnaA_QTL_ and anti-TnaA_XSPRING_ antibodies were used at 1:3000 and 1:250 dilutions respectively. Mouse anti-β-tubulin (E7, Developmental Studies Hybridoma Bank) and anti-actin (JLA20, Developmental Studies Hybridoma Bank) were used each at 1:3000 dilution. Secondary antibodies were anti-rabbit HRP goat IgG (H+L) (65–6129) and anti-mouse HRP goat IgG/IgA/IgM (H+L) (A10668) (Invitrogen).

To detect Hox proteins Ubx and Scr, we used monoclonal antibodies FP3.38 [18] for Ubx, and 6H4.1 [19] for Scr. To detect Osa we used monoclonal Osa 15A8 [20]. These three antibodies were purchased from Developmental Studies Hybridoma Bank. Secondary antibodies anti-rabbit and anti-mouse Alexafluor 568 goat (red), and anti-rat Alexafluor 594 (Invitrogen) were used for confocal microscopy.

### Protein extraction and analyses

Larval soluble protein extracts for Western analyses were obtained either by homogenizing whole larvae in lysis buffer (250 mM sucrose, 50 mM Tris pH 7.5, 25 mM KCl, 5 mM MgCl_2_, Complete protease inhibitor (ROCHE), 5 mM EDTA, 1% SDS) or by inverting the anterior part of half larvae according to Cunningham *et al*., (2012) [21] directions, where 10–20 third instar larvae were cut in half and inverted to remove trachea, gut and adipose tissue. In this case the remaining tissue including central nervous system, imaginal discs and salivary glands was homogenized in lysis buffer (PBS, 1% Triton X-100, 1 mM MgCl_2_, 5 mM EDTA and Complete protease inhibitor from ROCHE). Extracts obtained in either way were centrifuged at 10,000 g for 10 minutes at 4°C to remove cell debris. The proteins were separated by SDS-PAGE and electro-transferred onto nitrocellulose membranes for Western blot analyses. Immunoblots were done according to standard procedures and proteins of interest were detected with specific antibodies with the kits Supersignal West Pico, and Femto Chemiluminescent Substrates from Thermo Scientific, according to manufacturer’s instructions.

Quantification of TnaA isoforms in mutant genotypes was done by using the densitometry measurement tool from ImageJ (Fiji). Raw values were normalized according to the respective loading control in each lane, and final values were expressed as a percentage of protein relative to the one found in wild-type animals.

### Induction of mitotic clones

The *tna*^*1*^ allele recombined into an *FRT2A* chromosome (*tna*^*1*^ *FRT2A*) was a kind gift from J. A. Kennison. *tna*^*1*^ clones were induced either with the *hs-FLP* [22] or the *Ubx-FLP* [23, 24] drivers as FLPase sources.

To induce clones in imaginal discs with heat shock, we basically used the protocol reported by [25]. Briefly, clones were induced in the progeny of the cross *tna*^*1*^ *FRT2A/TM6B*, *Tb*, *Dr* X *hs-FLP*; +; *Ubi-GFPX2 FRT2A* that was set up at 25°C. Clones were induced in the progeny at 24 h after egg laying (AEL) with a single 37.5°C heat shock for 1 h. Some clones in haltere discs were induced in an egg collection of 4 h, applying a heat shock of 38.5°C for 1 h to the progeny at 9 h AEL.

We also used the *Ubx-FLP* [24], that induces recombination through a *Ubx* enhancer that is active in all imaginal discs (IDE, Imaginal Disc Enhancer), identified in the PBX-41 segment [23]. Crosses were set with *Ubx-FLP/*Y; *tna*^*1*^ *FRT2A/TM6B/+* X *+; Ubi-GFP FRT2A*, or males +; *FRT82B osa*^*308*^*/TM6B Dr*, *Tb* X *Ubx-FLP; +; FRT82B Ubi-mRFP* at 25°C. Animals with induced clones either with *hs-FLP* or with *Ubx-FLP* were kept at 25°C until they reached 110 to 115 h AEL, where discs were dissected for immunostaining with the antibodies of interest and observed using confocal microscopy as stated in the next section, or animals were allowed to reach the adult stage to estimate cuticular Hox loss-of-function phenotypes.

### Immunostaining of imaginal discs and cuticle preparations

Wing and haltere discs were obtained from male and female third instar larvae. Male first leg discs were isolated from sexed third instar larvae to study *Scr* expression. Immunostaining of imaginal discs were done as described by Blair (2000) [26] with some modifications. Briefly, imaginal discs were dissected in cold 1X PBS and fixed with 4% paraformaldehyde for 30 min at room temperature. Discs were washed with PBT (1X PBS with 0.2% Triton X-100), blocked for 1 h with 0.1% bovine serum albumine in PBST 1X with 250 mM NaCl at 4°C. Primary antibodies were added at appropriate dilutions overnight at 4°C, and the next day discs were washed with PBT. Secondary antibodies were added together with Hoechst (0.1 ug/ml) for 2 h at room temperature washed again with PBST and after removal of PBST, discs were mounted in 80% glycerol, 4% *n*-propyl gallate in 1X PBS and stored in darkness until observation in confocal microscopy.

Fluorescent images from immunostained imaginal discs, or for detection of Hox proteins or apoptotic cells (TUNEL assay), were acquired with an Olympus Inverted FV1000, or a 2P Upright confocal FV1000 confocal microscopes with a 20X 0.75 or 60X 1.3 numerical aperture objectives. Images were processed using ImageJ (Fiji) and Adobe Photoshop CS software.

Adult cuticle preparations were processed by standard procedures by boiling flies in 10% KOH, washed in distilled water, and mounting them in glycerol 50% to observe them in a Nikkon Eclipse E600 upright microscope equipped with an Amscope MU500 digital camera.

### Cell survival analyses

To assess the role of *tna* on cell survival, we compared the areas of both *tna*^*1*^*/tna*^*1*^ (GFP^-/-^), and *tna*^*+*^*/tna*^*+*^ twin-spot (GFP^+/+^) clones induced 24 h AEL. Images were analyzed with ImageJ (Fiji) to measure the area of 13 clones and their twin spots. A paired t-test was used to assess significant differences in their respective areas (P<0.05) (S1 Fig).

To determine whether *tna*^*1*^/*tna*^*1*^ genotype could cause cell death, we performed a TUNEL (Terminal deoxynucleotidyl-transferase-mediated dUTP Nick end Labeling) cell-death assay, with the *In Situ* Cell Death Detection kit TMR red, (Roche, cat. no. 12156792910), according to manufacturer instructions. Apoptotic cells in wing imaginal discs of genotype *MS1096-GAL4 UAS-ras*^*V12*^ *UAS-dlg*^*RNAi*^ were used as a positive control. For the cell death assay, imaginal discs were dissected, fixed and washed as for immunostaining. Confocal images were captured as stated in the previous section.

## Results

### *tna* is required at late phases of development

Pharates deficient in *tna* present cuticular Hox loss-of-function phenotypes [5]. To further study the effect of Hox gene expression in imaginal discs lacking TnaA at larval stages, we inspected with more detail a selected *tna* allelic set of combinations producing animals that die between the third instar larval stage and adulthood (Table 1). We used the *tna* alleles (Fig 1A) *tna*^*1*^ [5], *tna*^*5*^ that is a null allele [6], and *tna*^*EY22929*^ [12] that is a P{EPgy2} element insertion at 5’ end of *tna*. We combined each of these alleles between them, and with two chromosomal deficiencies that uncover *tna*, *Df(3L)378* and *Df(3L)vin2*. We also knocked down *tna* expression by inducing the expression of two different RNAi constructs, *tna*^*JF02536*^ from the TRiP collection [14] and *tna*^*GD12331*^ from the Vienna GD collection [13] directed towards different regions of *tna* mRNAs (Fig 1A).

**Table 1:** Survival of animals with *tna* mutant genotypes at pupal and adult stages.

	PUPAE		ADULTS
	Pupariation Rate^b^ (Observed/ Expected)^c^	Eclosion Rate^b^ (Eclosed/Total *tna/tna* Pupae)^d^	Survival^b^ (Observed/ Expected)^c^
Relevant Genotype^a^			
***tna***^***1***^			
*tna*^*5*^	147/495^e^ (30)	0/147 (0)	0/476^e^ (0)
*Df(3L)378*	115/392 (29)	0/115 (0)	0/383 (0)
*Df(3L)vin2*	84/215 (39)	0/84 (0)	0/174 (0)
***tna***^***5***^			
*Df(3L)378*	129/204 (63)	0/129 (0)	0/172 (0)
*Df(3L)vin2*	90/155 (58)	0/90 (0)	0/112 (0)
***tna***^***EY22029***^			
*tna*^*1*^	139/132 (105)^ns^	63/139 (45)	37/123 (30)
*tna*^*5*^	132/153 (86)^ns^	117/132 (89)^ns^	110/144 (76)^ns^
*Df(3L)378*	231/253 (91)^ns^	207/231 (90)^ns^	188/207 (90)^ns^
*Df(3L)vin2*	307/380 (81)^ns^	0/307 (0)	0/317 (0)

^a^. Relevant genotype shows the *tna* alleles in heteroallelic animals evaluated. The alleles carried by parental males are shown in bold at the top of each section.

^b^. Puparium formation, eclosion rate, and adult survival were evaluated in progeny from the same crosses in at least two independent replicas. Statistical significance in each case was determined with a χ^2^ or t-test (P<0.05) as stated in Material and Methods. Non-significant differences are indicated (ns).

^c^. The percentage (in parentheses) of *tna/tna* heteroallelic individuals (non-Tubby pupae or non-Sb and/or non-Dr adults) was calculated taking as 100% the *tna/*Balancer individuals in progeny (half of the Tubby pupae or half of the Sb and/or Dr adults).

^d^. Percentages of eclosed pupae (in parentheses) were calculated dividing the number of eclosed over the total number of *tna/tna* (non-Tubby) (non-eclosed plus eclosed) pupae.

^e^. The disparity between the numbers of expected pupae and adults (compare first and third columns) is caused because the balancer chromosomes from mothers and fathers have Tubby as a larval/pupal marker, and in adults one balancer chromosome carried Sb and the other one has Dr. It was not possible to distinguish which balancer chromosome carry each Tubby pupae counted, but one class carrying one of the parental balancer chromosomes is more lethal than the other. When divided by two, to calculate the expected *tna/tna* progeny we found a difference between the number of Tb eclosed pupae and Sb or Dr adult flies. e. g. first lane: Half of Tubby pupae = 495. Adults with Dr marker = 476.

**Fig 2:**
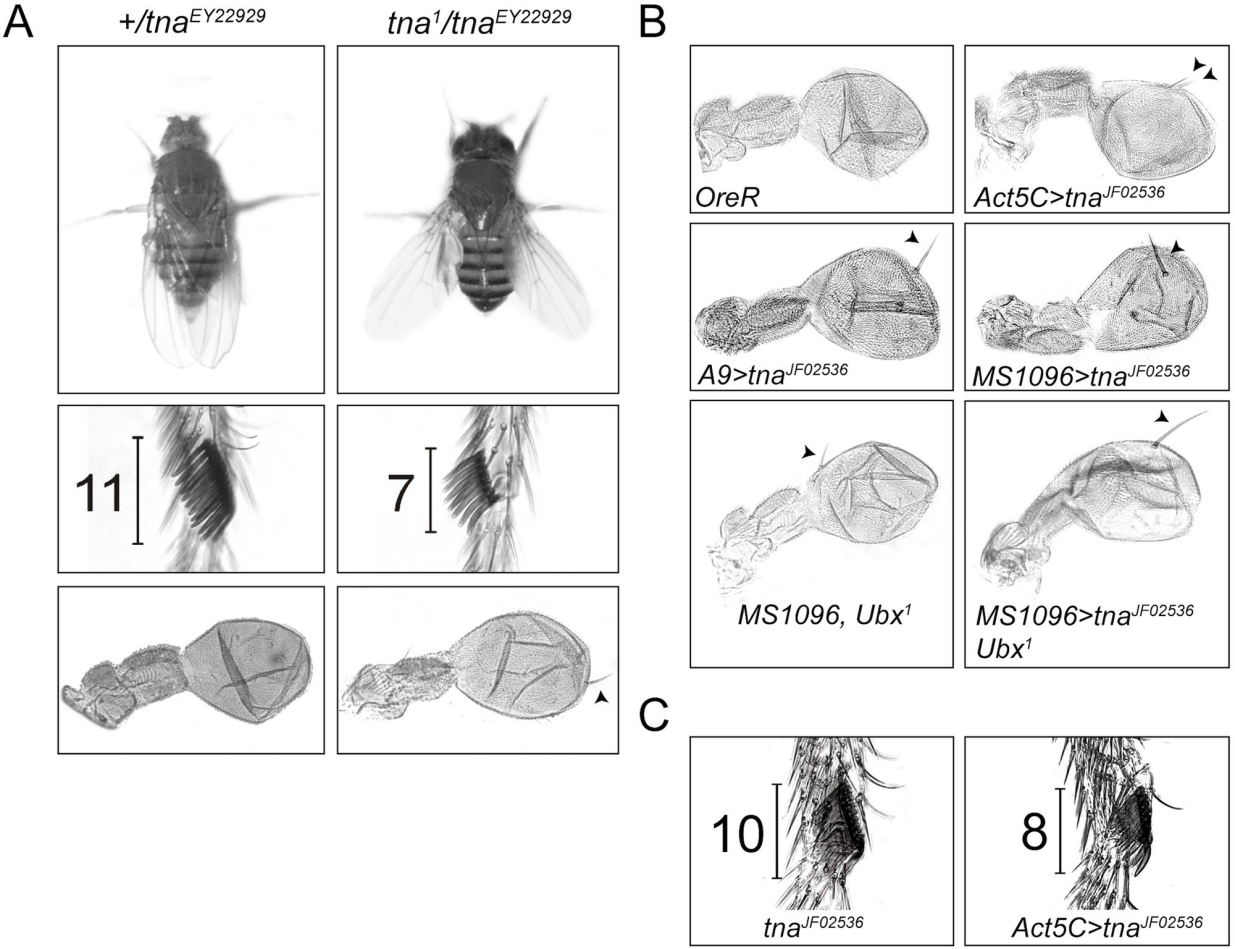
Hox transformations in *tna* mutant and *tna* knocked down adult animals. (**A**) Held-out wing (upper), reduction of the number of sex comb teeth in first leg of males (middle) and ectopic bristle in the haltere (lower), indicating loss-of-function of *Antennapedia* (*Antp*) *P2* promoter, *Scr*, and *Ubx* respectively, in *tna*^*EY22929*^/*tna*^*1*^ (right), compared to wild type phenotypes (left) in *tna*^*EY22929*^/+ flies. (**B**) Appearance of ectopic bristles in halteres indicates a haltere-to-wing transformation in flies expressing the RNAi produced by the *tna*^*JF02536*^ allele with different GAL4 drivers (*Act5C*, *A9*, *MS1096*) (right). Parental flies with the mentioned drivers (not shown) have halteres with wild-type phenotypes (*OreR* haltere in upper left picture). Note that the location of the ectopic bristle in animals with *tna* knockdown, depends on the location where the *tna* RNAi is directed. (**C**) Males with ubiquitous *tna* knockdown (*Act5C>tna*^*JF02536*^), do not survive to adulthood. Presented here is the first leg of a pupal male individual with a decrease in the number of sex comb teeth (right) compared to wild type (left). For percentages of these phenotypes see Table 2.

The genetic analyses we made involve the determination of pupal and adult survival of animals with heteroallelic *tna* combinations (Table 1). We evaluated the number of heteroallelic animals that reach the pupal stage, and of those we counted how many were able to eclose from their pupal cases. We found that *tna*^*1*^ is the strongest allele tested. This is expected because we have shown previously that *tna*^*1*^ is a dominant negative mutation [6]. Only between 30–40% of the expected animals with *tna*^*1*^ as one of the alleles in the genotype (together with *tna*^*5*^ or with *Df(3L)378* and *Df(3L)vin2*), reach the pupal stage. Close to 60% of the animals with *tna*^*5*^ (together with any of the two deficiency chromosomes) reach the pupal stage, dying before reaching adulthood. In contrast, 80–100% of the animals with *tna*^*EY22929*^ reach the pupal stage. None of the pupae with *tna*^*1*^ or *tna*^*5*^ were able to eclose from their pupal cases, with the notorious exception of combinations of these alleles with *tna*^*EY22929*^ allele where some animals survive until adulthood. 45–90% of animals with *tna*^*EY22929*^ eclose (with the exception of animals harboring also *Df(3L)vin2* that die as pupae), and from those 30–90% do reach adulthood. Of notice, all the adult animals of *tna*^*1*^*/ tna*^*EY22929*^ genotype present the held-out wings phenotype (Fig 2A) that was the base for the identification of *tna* as a *brm*-modifier in our original screen [5]. Adult flies with *tna*^*1*^*/ tna*^*EY22929*^, or *tna*^*EY22929*^*/Df(3L)378* genotypes, present Hox loss-of-function phenotypes such as loss of sex comb teeth in male first legs (*Scr*), and partial haltere to wing transformation (*Ubx*) (Fig 2A and Table 2).

**Table 2:** Hox phenotypes in adults with *tna* mutant genotypes, with *tna* knockdown, or where *tna*^-^ clones were induced in imaginal discs.

Relevant Genotype	*Antp P2* Held-out wings	*Ubx* Haltere to wing^a^	*Scr* < 9 teeth/sex comb^b^
*OreR*	0/72 (0)	0/72 (0)	0/48 (0)
*tna*^*EY22929*^	0/62 (0)	0/62 (0)	0/44 (0)
*tna*^*1*^*/tna*^*EY22929*^	54/54 (100)	15/54 (28)	30/39 (77)
*tna*^*EY22929*^*/Df(3L)378*	0/63 (0)	7/63 (11)	32/37 (87)
***tna* knockdown**^c^			
*Act5-GAL4*	0/85 (0)	0/85 (0)	0/43 (0)
*tna*^*JF25036*^	0/96 (0)	0/96 (0)	0/32 (0)
*Act5-GAL4>tna*^*JF25036*^	0/136 (0)	22/136 (16)	4/29 (14)
*A9-GAL4*	NA	0/110 (0)	NA
*A9-GAL4>tna*^*JF25036*^	NA	53/345 (15)	NA
*MS1096-GAL4*	NA	0/95 (0)	NA
*MS1096-GAL4; Ubx*^*1*^	NA	19/87 (22)	NA
*MS1096-GAL4>tna*^*JF25036*^	NA	12/152 (8)	NA
*MS1096-GAL4>tna*^*JF25036*^*/Ubx*^*1*^	NA	122/142 (86)	NA
**Adults from mitotic imaginal clones**^**NA**^			
*hs-FLP*; *tna*^*1*^ *FRT2A/Ubi-GFP FRT2A*			
No heat shock	0/47 (0)	0/47 (0)	0/49 (0)
Heat shock^d^	23/159 (15)	1/55 (2)	2/49 (4)
*Ubx-FLP; tna*^*1*^ *FRT2A*	0/29 (0)	0/29 (0)	0/37 (0)
*Ubx-FLP; tna*^*1*^ *FRT2A*/*Ubi-GFP FRT2A*	34/50 (68)	7/50 (14)	0/33 (0)

Percentages are in parentheses. NA is non applicable. The number of individuals showing the indicated Hox loss-of-function phenotypes is statistically significant (t-test, P<0.01, see Material and methods). Statistical test was not applicable (NA) for the evaluation of phenotypes of adults from mitotic imaginal clones induction because in principle it was not known how many clones were induced in each case.

^a^. Adult individuals with at least one partially transformed haltere.

^b^. Adult (*tna*^*1*^*/tna*^*EY22929*^) or pharate (*Act5-GAL4>tna*^*JF25036*^) males with less than 9 sex comb teeth per leg.

^c^. Flies expressing *tna* RNAi from *tna*^*JF25036*^ were raised at 28°C.

^d^. Heat shock was applied as established in Material and Methods at 24 AEL.

As *tna locus* harbors different transcripts that produce different isoforms, mainly TnaA_130_ and TnaA_123_, we were interested in correlate which of them was present in larvae of the genotypes studied. We performed Western analyses with anti-TnaA antibodies (Fig 1B) that detect both TnaA_130_ and TnaA_123_ isoforms (TnaA_XSPRING_) or TnaA_123_ preferentially (TnaA_QTL_) in protein soluble extracts from larvae of some of the genotypes tested and from its siblings that carried a wild type *tna* allele for comparison (Fig 1C–1F). We also tested animals with genotypes that included the *tna*^-^ *Df(3L)378* and *Df(3L)vin2* deficiency chromosomes (Fig 1D and 1F). Animals with combinations that include either one of these deficiencies, survive up to the third instar larvae stage in significant percentages (from 30–90% of the expected individuals, Table 1) and then die as pupae. This fact allows us to analyze protein extracts from these mutant third instar larvae, making it easier to determine which TnaA isoforms were affected specifically with the *tna* mutant alleles we were testing, given that combined with the deficiency chromosomes, these alleles would be the only source of TnaA. We also analyzed soluble extracts from larvae with *tna* knockdown by the expression of *tna*^*JF02536*^ at 28°C (Fig 1G).

We found that TnaA_130_ disappeared in all the mutant genotypes tested (Fig 1), particularly when one of the alleles is *tna*^*EY22929*^, while TnaA_123_ disappears (*tna*^*1*^/*tna*^*5*^, Fig 1C and 1H), or it is still detected at a much lower concentration than in a wild-type condition (see for example, *tna*^*1*^/*tna*^*EY22029*^, or *tna*^*EY22929*^/*Df(3L)378*, Fig 1E, 1F and 1H) with antibodies (TnaA_QTL_ and TnaA_XSPRING_). We also found that RNAi expression from *tna*^*JF02536*^ at 28°C, knocked down *tna* expression almost 90% (see ahead, Fig 1G). In particular, we compared *tna*^*EY22929*^/*Df(3L)vin2* and *tna*^*EY22929*^/*Df(3L)378* (Fig 1D and 1F) because they give totally different results regarding survival to adulthood (Table 1). While 90% of *tna*^*EY22929*^/*Df(3L)378* animals survive to adult stages, none of the *tna*^*EY22929*^/*Df(3L)vin2* animals survive to this stage. In contrast, animals from both genotypes reach the pupal stage with the difference that 100% of *tna*^*EY22929*^/*Df(3L)vin2* die before they eclose (Table 1). Western analyses are in agreement with these findings given that *tna*^*EY22929*^/*Df(3L)378* larvae still present some detectable levels of TnaA_123_ protein (Fig 1F and 1H), that may account for their 90% survival to adulthood, meanwhile in *tna*^*EY22929*^/*Df(3L)vin2* larvae, TnaA_123_ isoform is barely detectable (Fig 1D and 1H) and they present 100% of lethality in adult stages.

These results indicate two findings. First, the presence of TnaA_130_ is not required for survival to adulthood, because animals lacking it, reach this stage (*tna*^*1*^/*tna*^*EY22029*^, and *tna*^*EY22929*^/*Df(3L)378*). We noticed that although *tna*^*1*^*/tna*^*EY22929*^ animals present a slightly higher amount of TnaA_123_ than the one found in *tna*^*EY22929*^*/Df(3L)378* animals, the latter ones have a better adult survival (90% compared to 30% of *tna*^*1*^*/tna*^*EY22929*^). This difference may be due to the fact that *tna*^*1*^ is a dominant negative [6]. The second finding is that the P{EPgy2} element insertion in the *tna*^*EY22929*^ allele, is affecting the expression of tna-RA transcript that would be encoding TnaA_130_. Moreover, one of our hypotheses was that TnaA_123_ could originate from TnaA_130_ processing [6], but with these data, we support the hypothesis that TnaA_123_ and TnaA_130_, are translated from different transcripts, being those tna-RD and tna-RA respectively.

For *tna* knockdown, two *UAS-RNAi* constructs, *tna*^*JF02536*^ and *tna*^*GD12331*^, were expressed at 18, 25 and 28°C with the ubiquitous driver *Act5C-GAL4*. A higher percentage of lethality is observed by increasing *tna* RNAi expression at higher temperatures (Table 3). RNAi expression from either *tna*^*JF02536*^ or *tna*^*GD12331*^ alleles caused lethality of pupae and pharates, being males more sensitive than females. *tna*^*JF02536*^ induction caused more lethality than *tna*^*GD12331*^ induction and then it was further characterized. The effectiveness of the interference, caused by the expression of the RNAi from *tna*^*JF02536*^, was confirmed by the low protein levels found in these larvae (Fig 1G). Females from these experiments reached adulthood in higher percentages than males, but they die within 10 days after eclosion (Table 3). As for *tna*^*1*^*/ tna*^*EY22929*^ animals, knocking down *tna* through RNAi, result in *Ubx* and *Scr* loss-of-function phenotypes at, albeit low, measurable penetrance (Fig 2B and 2C and Table 2). *Act5C-Gal4* is a strong ubiquitous driver. To study the effect of knocking down *tna* in a restricted spatial domain within the haltere, we induced the expression of *tna* RNAi to the dorsal region of the haltere pouch with the GAL4 drivers *MS1096* and *A9* (see Material and methods). The animals from these experiments showed ectopic bristles located mainly in the dorsal region of the haltere capitellum. To test whether this partial transformation was caused by a reduction in *Ubx* expression, we tested whether *tna* knockdown (by expressing RNAi from *tna*^*JF02536*^), enhances the loss-of-function *Ubx* phenotypes observed in animals with the null *Ubx*^*1*^ allele [27]. The result of this genetic combination (Table 2) was the appearance of a single ectopic bristle in the dorsal distal pouch region of the transformed halteres (Fig 2B). *tna* knockdown enhanced the penetrance and the expressivity of the *Ubx*^*1*^ phenotype (from 22 to 86%, observed as a higher percentage of halteres with a single larger bristle than the one present in halteres of *Ubx*^*1*^/+ halteres genotype) (Table 2, Fig 2B).

**Table 3:** Survival of flies with *tna* knockdown.

Relevant Genotype		Survival to adulthood of flies bred at:		
		18°C	25°C	28°C
*Act5C-GAL4/+*^a^				
*tna*^*GD12331*^	F^b^	95/105 (91)^ns^	81/127 (64)	4/98 (4)
	M^b^	107/114 (94)^ns^	46/97 (47)	0/112 (0)
*tna*^*JF02536*^	F	81/115 (70)	107/222 (48)	5/125 (4)
	M	1/109 (1)	1/183 (1)	3/169 (2)

^a^. *Act5C-GAL4* driver directs ubiquitous expression of *tna*^*GD12331*^ or *tna*^*JF02536*^ (Fig 1A).

^b^. Female (F) or male (M) adult progeny expressing the indicated RNAi. The proportion indicates the survival of adult flies expressing the indicated RNAi with respect to the survival of the healthier class in the cross (which is not expressing the RNAi) at the indicated temperature. Percentages of each proportion are in parentheses. Note that survival is more affected in males than in females and it diminishes by increasing the breeding temperature, where RNAi expression is increased. Data are from three independent crosses for each genotype, n = 3, and are statistically significant (χ^2^ test, P<0.01), except the ones labeled ns (non-significant).

In conclusion, reduction of TnaA dosages caused by different *tna* null or hypomorphic alleles or by knocking down its expression, causes different grades of lethality through development and, when animals are able to form cuticles, homeotic defects caused by loss-of-function of several Hox genes are evident at different extents (penetrances and expressivities).

### *tna*^*1*^ mitotic clones induced either at embryonic or larval stages survive normally

**Fig 3:**
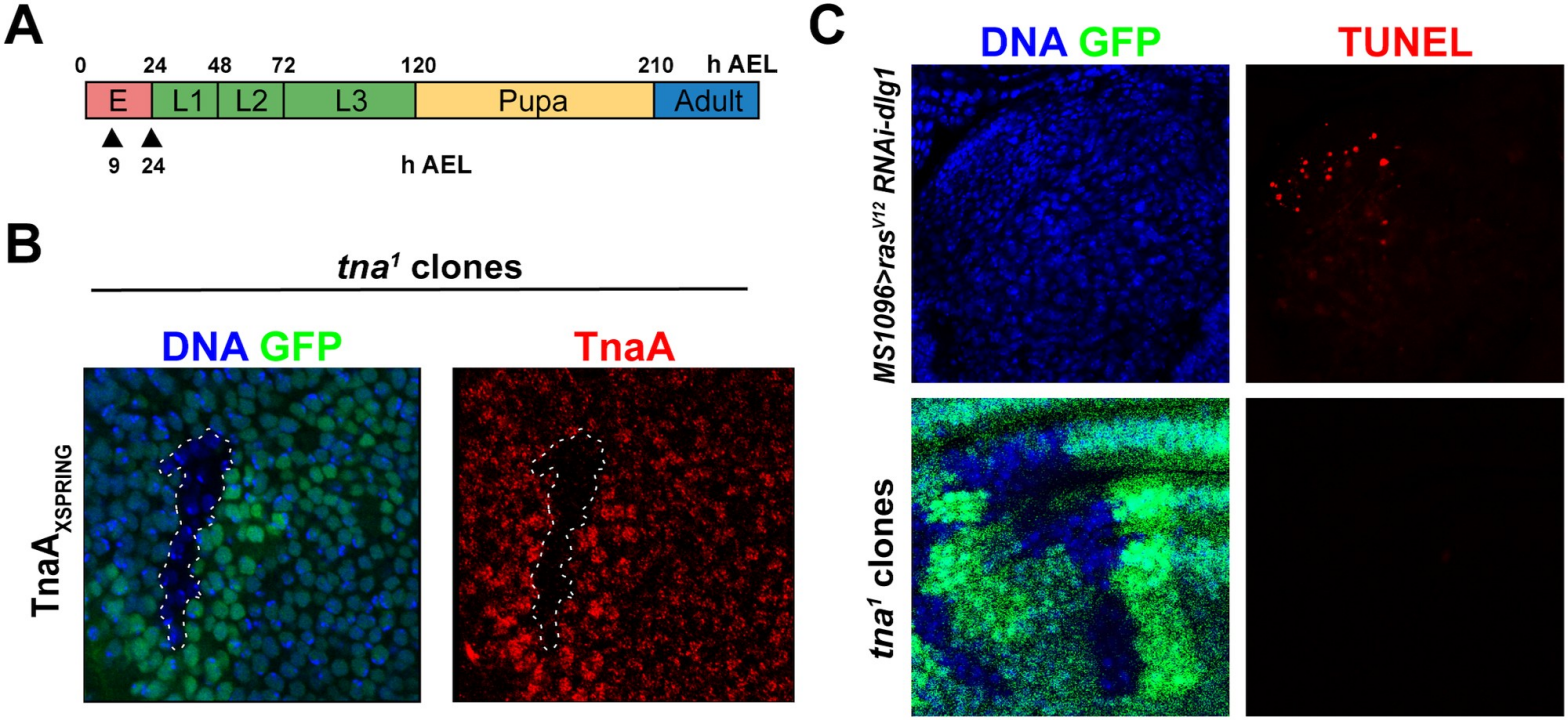
Induction of TnaA defective mitotic clones in third instar imaginal discs at different times of development does not cause cell death. (**A**) Timeline of heat shock pulses (black triangles After Egg Laying, AEL) applied to induce mitotic recombination in animals bearing *hs-FLP* (*hs-FLP*; +; *tna*^*1*^ *FRT2A/Ubi-GFPX2 FRT2A*). (**B**) Immunostaining of TnaA with the TnaA_XSPRING_ antibody in a wing disc where mitotic clones were induced. DNA was stained with Hoechst (blue) to show nuclear presence. GFP (green) marks the *tna*^*+*^/*tna*^-^ and that did not recombine (medium green intensity), and the *tna*^*+*^/*tna*^*+*^ (strong green intensity) cells result of the recombination event. GFP^-^ marks the *tna*^-^/*tna*^*-*^ clone, as corroborated by the absence of TnaA immunostaining (red). (**C**) TUNEL death assay (red) performed in imaginal discs with *tna*^*-*^ clones (GFP^-^), Hoechst (blue), (*tna*^*+*^ cells are GFP^+^, green) (lower panels). As a positive cell death control (red), apoptotic cells were detected in larval discs of the genotype *MS1096-GAL4 UAS- ras*^*V12*^ *UAS-dlg*^*RNAi*^ (see text) (upper panels). Note also that in general here and in the next figures, the number and size of the *tna*^*-*^/*tna*^*-*^ (GFP^-^) cells compared to the ones in the *tna*^*+*^/*tna*^*+*^ (GFP^+^) clone seems to be similar, showing that *tna*^*-*^/*tna*^*-*^ cells do not present an obvious defect.

**Fig 4:**
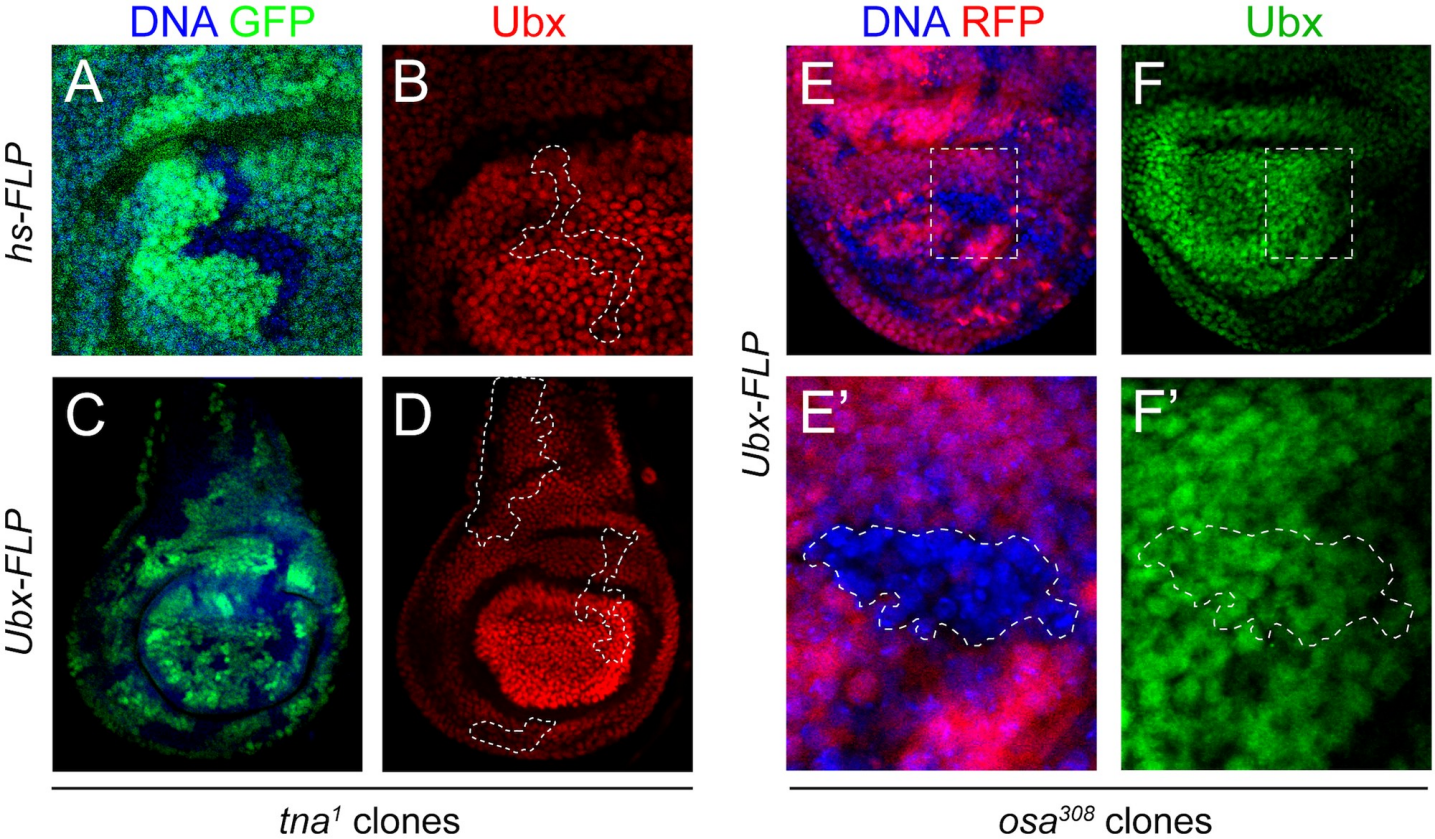
Ubx protein is present in TnaA or Osa defective mitotic clones in haltere discs. TnaA (GFP^-^ in **A** and **C** and Fig 3B) and Osa (RFP^-^ in **E** and **E’**, and S3 Fig) defective mitotic clones induced by expressing FLPase either from *hs-FLP* (**A** and **B**) or *Ubx-FLP* (**C**, **D**, **E’**, and **F’**) in haltere imaginal discs. Almost the whole disc is shown in **C** and **D** to note that the absence of TnaA (GFP^-^) in any region of the disc does not affect the presence of Ubx, which is observed in a wild-type pattern. Ubx protein was immunostained with monoclonal antibody FP3.38 [18] (red signal in **B**, and **D**, and green in **F**, and **F’**). Note that no decrease or absence of the Hox protein Ubx is observed in any of the TnaA^-^ or Osa^-^ clones (labeled with pointed white shapes).

Previously we showed that loss of maternal *tna* function is completely rescued paternally and loss of both maternal and zygotic functions caused lethality primarily at the third larval instar [5]. To further investigate the role of *tna* in imaginal disc cells, we generated *tna* deficient mitotic clones. We recombined the lesion in *tna*^*1*^ from a *tna*^*1*^*FRT2A* chromosome (gift from J. A. Kennison, see Methods), with the FLP/FRT system, expressing the FLPase either by heat shock at different times of development (Fig 3A), or under the control of an enhancer active in all discs (Imaginal Disc Enhancer, IDE) [24], identified in the *Ubx* PBX-41 segment [23]. With both methods, we were able to get *tna*^-^ GFP^-^ clones that we checked by immunostaining with the anti-TnaA_XSPRING_ antibody (Fig 3B). We were able to induce *tna* mutant clones in all the discs and we found in general that these clones survive well in all types of imaginal discs. We did not notice any change in the size or in the frequency of appearance of the GFP^-^ *tna*^-^ clones compared to GFP^+^ *tna*^+^ twin spots in discs (GFP panels in Fig 3B, 3C and S1 Fig), with the exception of clones in haltere discs, where we noticed often that the GFP^+^ twin spot was slightly larger than the *tna*^-^ GFP^-^ cells (e. g. GFP panel in Fig 4A). Neither, we observed any effect in cells in the vicinity within the border of *tna*^-^ clones. We also evaluated cell death by TUNEL in clones made in the wing disc (Fig 3C), finding that there was no difference in survival between *tna*^*1*^*/tna*^*+*^, *tna*^*+*^*/tna*^*+*^ cells, and *tna*^*1*^*/tna*^*1*^ mutant clones, while we detect cell death in the dorsal pouch of wing discs where apoptosis and overgrowth are induced by disrupting apical-basal cell polarity (Fig 3C) as reported by [28].

Thus, according to the results of these genetic and immunostaining assays, in general *tna* does not seem to influence cell survival, or cell number or size in imaginal discs.

### Influence of TnaA in Hox expression in larval imaginal discs

Our next goal was to study Hox expression in *tna*^*-*^ cells in the region of imaginal discs that will become the adult cuticle where loss-of-function homeotic transformations have been characterized. In particular, we focused on *Ubx* and *Scr* expression that is affected in pharates with *tna* mutant genotypes [5], in adults with the genotype *tna*^*1*^*/ tna*^*EY22929*^ (Fig 2A, Table 2), or where *tna* expression has been knocked down through the expression of the RNAi from *tna*^*JF02536*^ (Fig 2B and 2C, Table 2).

In a wild-type haltere disc, *Ubx* is expressed strongly in the pouch (the region that will become the haltere of the adult fly), being the most prominent the posterior compartment [29] (see for example, Fig 4D). In these regions, Ubx represses several genes that direct wing development [30]. The halteres from the animals with the *tna* mutant aforementioned genotypes, present a mild haltere-to-wing transformation indicated by the presence of ectopic bristles in the haltere capitellum (Fig 2A and 2B). This resembles a loss-of-function phenotype of *Ubx* [30]. Taking into account these observations, we made *tna*^*-*^ (*GFP*^*-*^) clones in haltere discs by inducing recombination either with an *hs-FLP* (Fig 4A), or with the *Ubx-FLP* (Fig 4C). We dissected haltere discs from animals where clones were induced, and they were immunostained for Ubx (Fig 4B and 4D, red signal). Other animals from the same experiments were allowed to reach the adult stage to evaluate the presence of the ectopic bristles in the haltere (*Ubx*^*-*^ phenotype) (Table 2). We found that the *Ubx*^*-*^ phenotype was present in the halteres of adult flies where clones were induced (2% to 14% when recombination was induced either with heat shock or with the Ubx-FLPase, respectively, Table 2). The presence of this transformation corroborate that cells producing the mutant phenotype in the haltere disc were hit by the recombination event. We tried several protocols to induce *tna*^*-*^ clones at different moments of development (with heat shock at 9 or 24 h AEL as stated in Fig 3A) (Fig 4A) or by getting *tna*^*-*^ clones in several regions of the disc (by driving recombination with the *Ubx-FLP* that is expressed widely in imaginal discs, Fig 4C). We specially looked for clones in the region of the haltere disc that produces the ectopic bristle in the adult halteres in *tna* mutant animals from the same experiments (Table 2), and we could not detect a reduction Ubx (Fig 4B and 4D) in any of the *tna*^*-*^ (*GFP*^*-*^) clones induced with any of the treatments tested.

**Fig 5:**
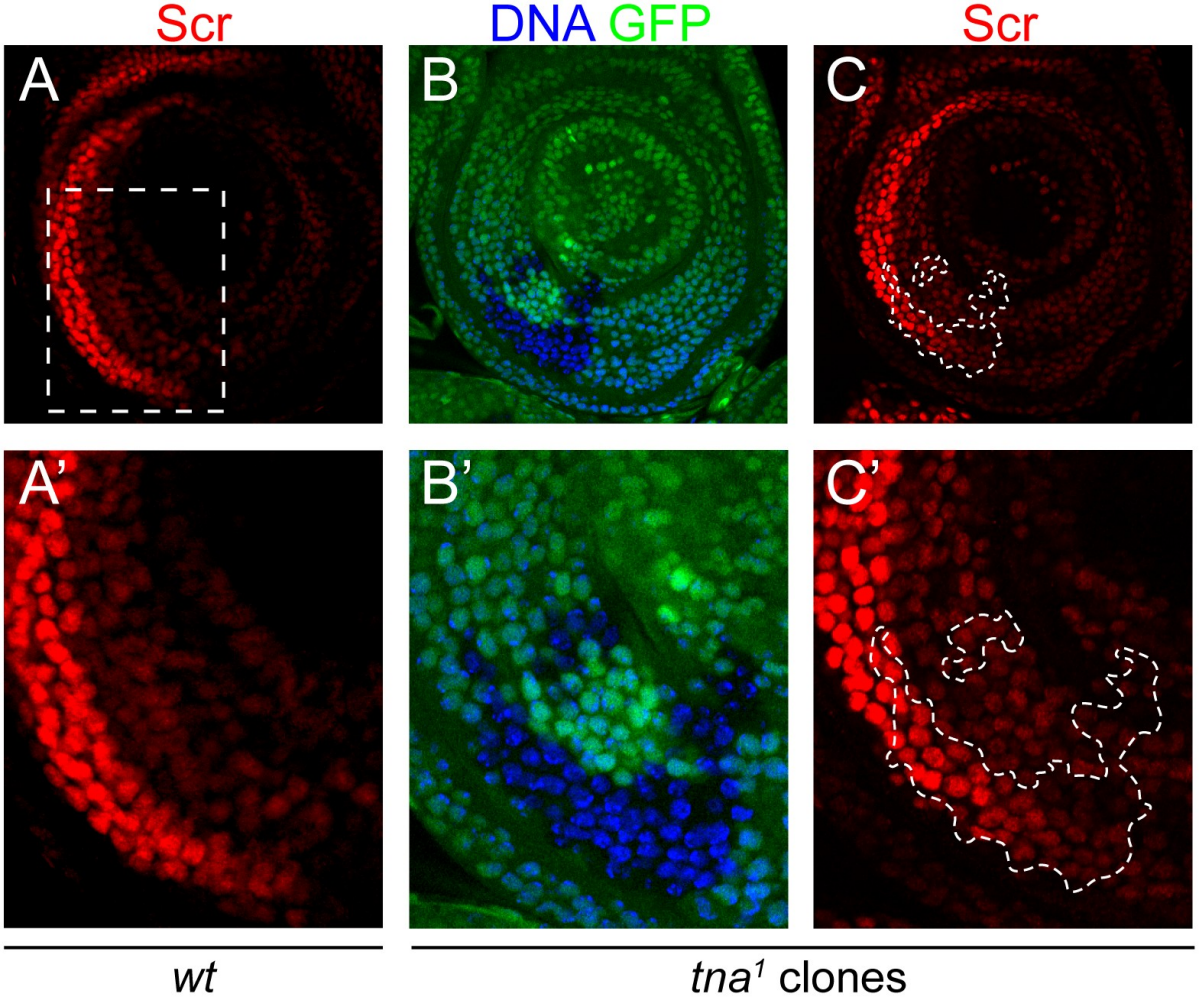
Scr proteins are present in TnaA defective mitotic clones in prothoracic T1 leg imaginal discs. Scr immunostaining pattern in wild type *OreR* prothoracic leg discs (**A**). The disc region from where the sex combs in the first leg are originated in the male is labeled by a pointed rectangle (**A**) and amplified in **A’**. TnaA (GFP^-^ in **B**-**B’** and Fig 4B) defective mitotic clones induced by expressing FLPase from the *hs-FLP* chromosome in T1 leg imaginal discs. Scr protein was immunostained with the monoclonal antibody 6H4.1 [19] (red signal in **A-A’**, and **C-C’**). Note that no decrease or absence of Scr is observed in the TnaA^-^ clones (labeled with pointed white shapes **C**, and amplified in **C’**).

*Scr* is normally expressed in the prothoracic leg imaginal disc mostly in a crescent-shaped region in the anterior half of the disc, and in marginal regions near the disc stalk, specially on the posterior side [19] (for example, Fig 5A). *tna* mutant adults (e. g. *tna*^*1*^*/tna*^*EY22029*^) with decreased TnaA levels (Fig 1E), present *Scr* loss-of-function phenotype [5] (Fig 2A, and Table 2 this work). This phenotype is observed in adult males as a reduction in the number of sex comb teeth in the prothoracic T1 first leg. We immunostained for Scr, leg discs of *tna*^*1*^*/tna*^*EY22029*^ animals finding that decreased TnaA level does not affect immunostaining of Scr (S2 Fig).

Therefore, we generated *tna*^*-*^ (*GFP*^*-*^) clones in leg discs, inducing recombination by expressing the FLPase under the control of a heat shock promoter (Fig 5B and 5B’) (see Material and methods). Some animals were allowed to reach the adult stage, finding that 4% of the first leg of males analyzed have less than nine sex comb teeth (compared to a mean of 10.5 teeth per comb in wild type), phenotype caused by *Scr* loss-of-function (Table 2). *tna*^*-*^ (*GFP*^*-*^) clones from leg discs of animals of the same experiment were immunostained for Scr (Fig 5C and 5C’, red signal) and, as for the *tna*^*1*^*/tna*^*EY22029*^ discs, we could not find any *tna*^*-*^ clone where the signal of Scr immunostaining was reduced.

Osa is a subunit of the BRAHMA chromatin remodeling complex BAP. The *osa* gene interacts strongly with *tna* in a genetic assay of Hox gene expression in pharates and adults [5]. Thus, we also made *osa*^*-*^ (RFP^-^) clones in haltere (Fig 4E and 4E’, and S3 Fig) discs by inducing recombination of the strong loss-of-function *osa*^*308*^ allele [31]. In these Osa^-^ clones, as in the ones for *tna*, Ubx levels, estimated by immunostaining are not affected (Fig 4F and 4F’ green signal), indicating that the requirement of Osa, in the majority of these cells, is not essential for keeping the levels of these Hox proteins.

The penetrance of the *Ubx* and *Scr* loss-of-function phenotypes in animals derived from the experiments where the *tna*^*-*^ clones were induced, was very low (in the best of cases, 14% of halteres with an ectopic bristles, when *tna*^*-*^ clones were induced with Ubx-FLPase, Table 2), and we wondered whether we were not being able to detect by immunostaining the specific cells affected in the haltere or leg discs that would give origin to the transformed tissue in each case (the ectopic bristle found in the transformed halteres or the reduction of the number of sex comb teeth in the first leg of males, Table 2). Thus, we studied the effect of *tna* mutations in a derepressed homeotic background caused by loss of function of Polycomb.

**Fig 6:**
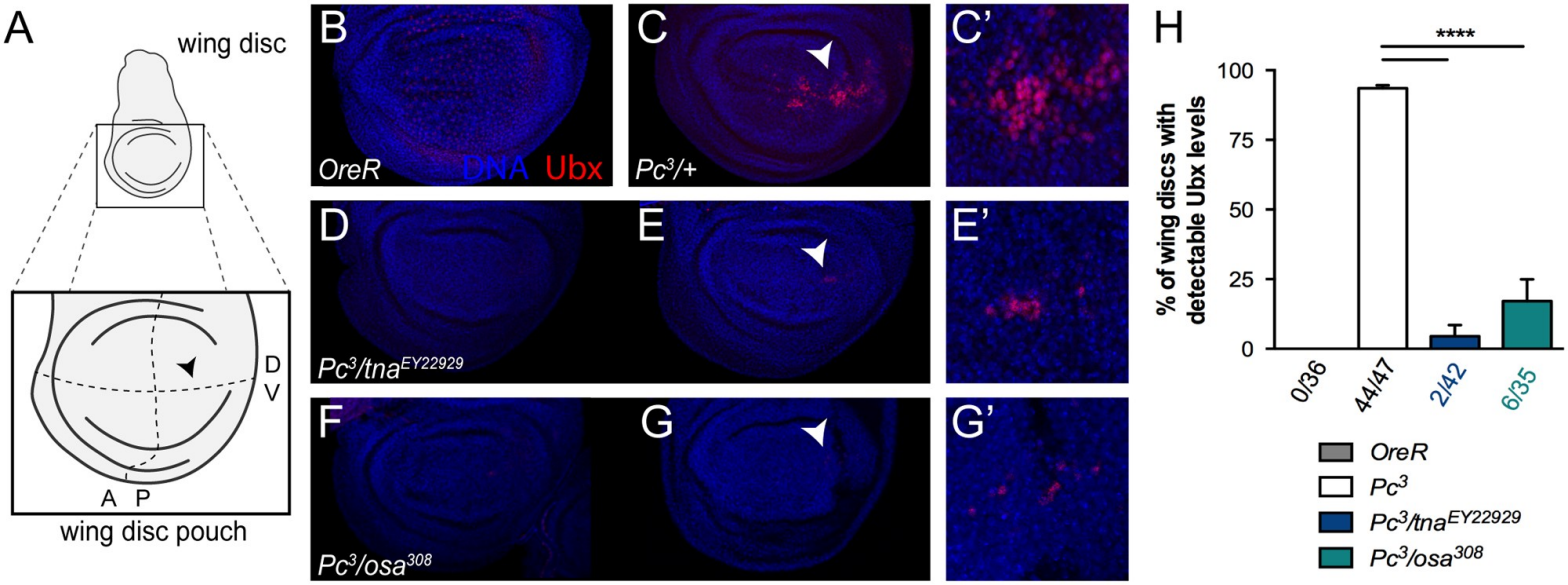
TnaA and Osa are necessary for ectopic presence of Ubx protein in wing *Pc*^*3*^ imaginal discs. (**A**) Wing disc organization. The posterior dorso-ventral margin is indicated (black arrowhead). Immunostaining of Ubx protein (red) with the FP3.38 [18] antibody in wild type *OreR* (**B**), *Pc*^*3*^ (**C**, and **C’**), *Pc*^*3*^/*tna*^*EY22929*^ (**D, E**-**E’**), and *Pc*^*3*^/*osa*^*308*^ (**F, G-G’**) wing discs. Discs were also stained with Hoechst to observe nuclei (blue). The region amplified in **C’**, **E’** and **G’** is labeled in **C**, **E** and **G** (white arrowheads). (**H**) Quantification of wing discs with positive Ubx immunostaining (Ubx^+^). The number of Ubx^+^/Total wing discs counted is indicated at the *x*-axis of the graphic. At least 40 discs of each genotype are counted derived from at least three independent replicas. There is statistical significance (t-test, P<0.05) in the proportions of discs with detectable Ubx among different genotypes (bottom) are indicated with an asterisk (*). Note that ectopic Ubx expression in *Pc*^*3*^ wing discs (**B, B’**) is suppressed by *tna* (**C-D’**) or *osa* (**E-F’**) haploinsufficiency.

**Fig 7:**
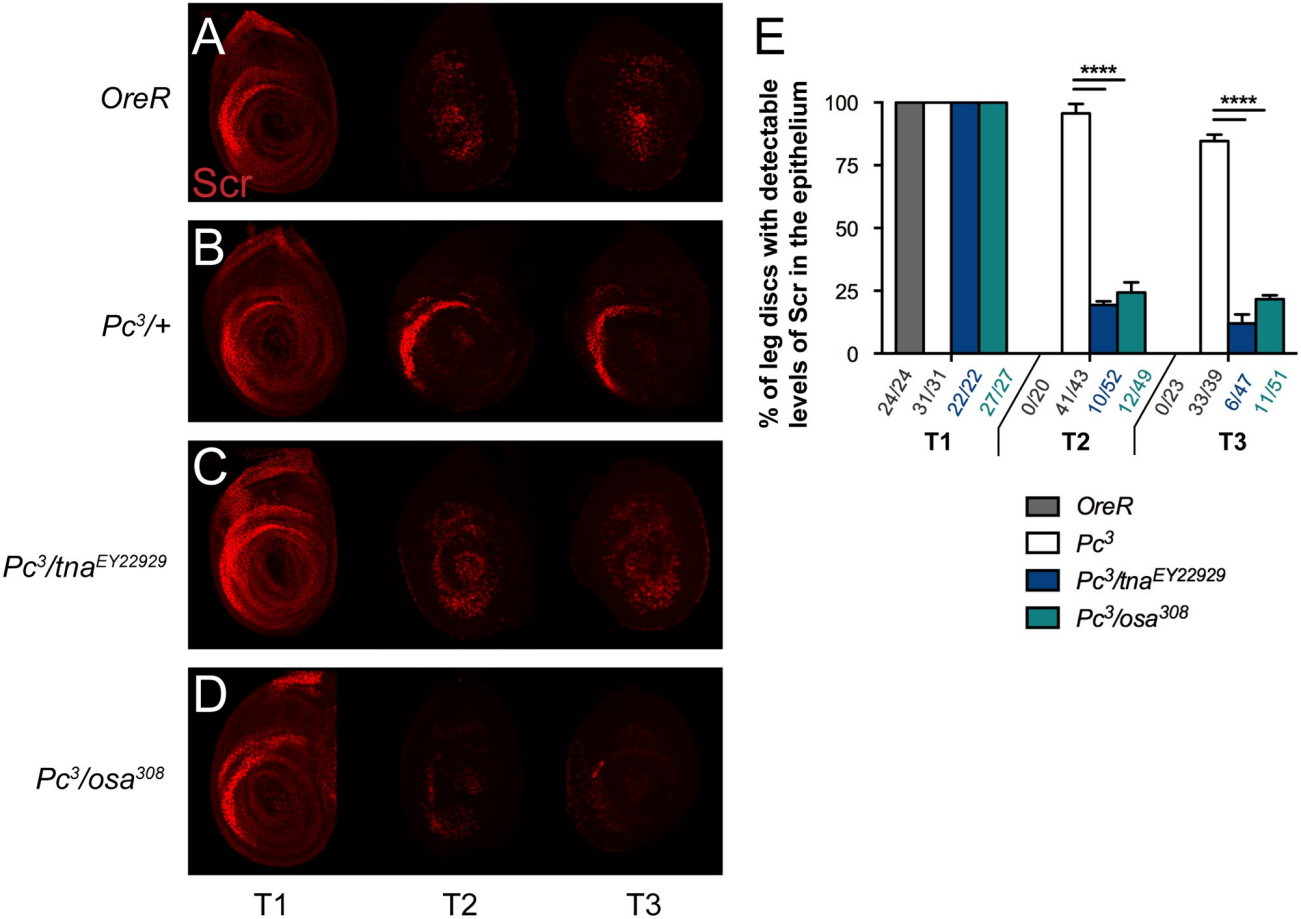
TnaA and Osa are necessary for ectopic presence of Scr protein in T2 and T3 *Pc*^*3*^ leg imaginal discs. Immunostaining of Scr protein (red) with the 6H4.1 [19] antibody in wild type *OreR* (**A**), *Pc*^*3*^ (**B**), *Pc*^*3*^/*tna*^*EY22929*^ (**C**), and *Pc*^*3*^/*osa*^*308*^ (**D**) T1-3 leg discs. (**E**) Quantification of haltere discs with positive Scr immunostaining (Scr^+^). The number of Scr^+^/Total haltere discs counted is indicated at the *x*-axis of the graphic. At least 40 discs of each genotype are counted derived from at least three independent replicas. Significant differences (t-test, P<0.05) in the proportions of discs with detectable Ubx among different genotypes (bottom) are indicated with an asterisk (*). Genotypes of discs counted are indicated in the bottom. Note that ectopic Scr expression in *Pc*^*3*^ T2 and T3 leg discs (**B**) is suppressed by *tna* (**C**) or *osa* (**D**) haploinsufficiency.

*Ubx* is normally expressed in the haltere disc while in the wing disc (Fig 6A, shows wing disc organization), *Ubx* expression is observed only in discrete areas such as the peripodial membrane [18] and it is not expressed in the epithelia. *Scr* is normally expressed in the first prothoracic leg imaginal disc (T1), but not in the second and third thoracic leg discs (T2 and T3) (Fig 7A). *Ubx* and *Scr* expression is derepressed in imaginal discs of animals harboring PcG loss-of-function mutations such as *Pc*^*3*^ (Figs 6C, 6C’ and 7B) [32, 33]. Derepression of both, *Ubx* (compare Fig 6B, 6C and 6C’) and *Scr* (compare Fig 7A and 7B) can be observed in wing, T2 and T3 leg discs of *Pc*^*3*^ heterozygote animals respectively. *tna* mutations suppress derepression of both Hox genes evaluated in cuticles from *Pc*^*3*^ pharate animals [5]. To investigate how *tna* influences *Ubx* and *Scr* expression in this context, we immunostained Ubx and Scr proteins in imaginal discs derived from *Pc*^*3*^ (Figs 6C, 6C’ and 6H and 7B and 7E respectively) and *Pc*^*3*^/*tna*^*EY22929*^ (Figs 6C, 6D, 6D’ and 6G and Fig 7C and 7E respectively) animals. As expected, we found ectopic production of both Ubx [94% (44/47) of *Pc*^*3*^ wing discs with Ubx signal, Fig 6B, 6B’ and 6G] and Scr proteins [93 (40/43) and 85% (33/39) of T2 and T3 *Pc*^*3*^ leg discs respectively with Scr signal, Fig 7B and 7E], while in discs from *Pc*^*3*^/*tna*^*EY22929*^ animals, *tna* haploinsufficiency suppresses close to 95% (2/42) *Ubx* ectopic expression (Fig 6D and 6H) and 83 to 87% *Scr* ectopic expression (9/52, and 6/47, positive Scr T2 and T3 immunostained leg discs, respectively) (Fig 7C and 7E). In a few cases [6% (2/42), Fig 6E and 6H], *Ubx* suppression was not total. A few cells remain that still have detectable Ubx immunostaining signal, (Fig 6E and 6E’). In the case of *Scr*, the suppression was clearly observed in both *Pc*^*3*^/*tna*^*EY22929*^ T2 and T3 leg discs (Fig 7C, in comparison to Fig 7B and 7E), while *Scr* expression in T1 leg disc looks normal (Fig 7 compare Fig 7A or 7B and 7C, see also Fig 7E).

In the same way, we tested whether *osa* haploinsufficiency was able to suppress Hox protein immunostaining in *Pc*^*3*^ discs. Indeed, we found that ectopic expression of *Ubx* (Fig 6B) and *Scr* (Fig 7B) in *Pc*^*3*^ wing discs was almost totally suppressed when a copy of *osa*^*308*^ was introduced (*Pc*^*3*^/*osa*^*308*^ discs) [83% (6/35) of suppression for Ubx, Fig 6F–6H, and 75% (12/49), and 78% (11/51) Scr suppression in T2 and T3 leg discs respectively, Fig 7D–7E].

In summary *tna* and *osa* are required to finely tune Hox expression and these subtle differences are not observed in the endogenous regions of Hox expression in the imaginal discs. A different situation is observed when *Ubx* and *Scr* expression is forced out of these regions and then, the requirements of *tna* and *osa* genes are revealed by the strong suppression of the ectopic Hox expression when these TrxG genes are inactivated.

## Discussion

In this work we studied the role of the TrxG gene *tna* on Hox gene expression in larval imaginal discs. First, we characterized the production of TnaA isoforms in different *tna* mutant genetic backgrounds. We also analyzed the Hox loss-of-function phenotypes present in adults with some of these *tna* mutant backgrounds. We found that the TnaA_123_ isoform is essential for larval, pupal, and adult survival. In contrast, we found, through mitotic clonal analyses, that *tna* is not required for individual cell survival in imaginal discs. Neither, we found decreased Hox expression in these *tna*-defective imaginal cells, although adult animals derived from these experiments do present the already characterized Hox loss-of-function phenotypes. We found that *tna*-defective function suppresses ectopic Hox expression in imaginal discs in a *Pc*-defective background. indicating that *tna* is a fine modulator of Hox gene expression. Below we discuss some possible mechanisms to explain how *tna* might be implicated in the expression of Hox and other genes.

### TnaA isoforms have dedicated functions related to the survival of post-embryonic stages

TrxG genes comprehend a functional diverse group that include among others, regulators of transcriptional initiation and elongation to maintain developmental gene expression (recently reviewed in [1]). *tna* encodes a group of proteins present in multicellular organisms, with a zinc SP-RING finger, characteristic of a type of SUMO E3-ligases. Besides the inherent complexity of the TrxG genes, biochemical studies are revealing that as *tna*, some genes of the group, encode several protein isoforms that may have dedicated functions. Up to date, we have detected at least three different protein products (two of them being TnaA_130_ and TnaA_123_) that may be the result of the expression from different promoters, alternative splicing, or post-translational modifications ([6] and this work). We have characterized that in embryos, TnaA_130_ is cytoplasmic, while TnaA_123_ is mainly nuclear [6]. One of the questions derived from this evidence is to determine whether or not these different isoforms are equivalent in function. In this work, we characterized the effect of reducing the main TnaA proteins (TnaA_130_ and TnaA_123_) on the expression of the Hox genes *Ubx* and *Scr* in imaginal discs. Through analyses of, first, the lethality shown by *tna* mutant animals that die since larval and pupal stages, second, the presence of homeotic transformations in the survivors with some of these genotypes, and third, the protein characterization produced by animals with different *tna* mutant alleles, we were able to make the following observations discarding previous hypotheses. TnaA_123_ is not a processing product of TnaA_130_. We can eliminate TnaA_130_ and still be able to observe TnaA_123_. TnaA_130_ does not affect the organism survival significantly, while TnaA_123_ must be at least detectable, to allow animals to reach the adult stages revealing for the first time a dedicated function for this isoform.

What proved to be more difficult was to determine whether the larval or pupal lethality in these *tna* mutant animals, was caused by a problem in Hox gene expression in imaginal discs at these developmental stages. Nevertheless, adult cuticles of *tna* mutant survivors do show Hox loss-of-function phenotypes. Third instar larvae with stronger *tna* alleles show no detectable TnaA_130_ and TnaA_123_ isoforms and die as late larvae or early pupal stages. In that sense, from these experiments we cannot discard the possibility that both, TnaA_130_ and TnaA_123_, could be contributing for proper Hox expression.

It is probable that TnaA may be required in two phases during development. In the first phase, the maternal deposition of TnaA might be important to establish the early chromatin landscape for Hox gene expression, in a similar way as the TrxG gene *Utx*. The Utx protein (Ubiquitously transcribed tetratricopeptide repeat protein X chromosome) is a demethylase of the lysine 27 of histone H3 deposited by the PcG [34, 35]. Animals without both, maternal and zygotic *Utx*, die as larvae and do not maintain Hox expression, attributed to the fact that some cells cannot initiate the maintenance of Hox gene expression at early stages during the onset of zygotic gene transcription [34, 35]. Animals carrying maternal but no zygotic Utx reach adulthood, have weak loss-of-function phenotypes of diverse Hox, and die just after eclosing, revealing the Utx requirement for viability [34, 35].

Previously, we showed that loss of maternal *tna* function is completely rescued paternally and loss of both maternal and zygotic functions caused lethality primarily at the third larval instar [5]. It will be important to determine whether depleting TnaA at early stages could result in a reduction in Hox expression. In this work we show that *tna* is required for viability at larval, pupal and adult stages, and for ectopic Hox expression in imaginal discs. Is *tna* a gene necessary to initiate the maintenance of Hox gene expression as *Utx*? It is probable that *tna* could participate in this mechanism and later on for viability. This would explain the weak Hox phenotypes and the lethality at late developmental stages of *tna* mutant animals, resembling the ones observed in individuals lacking *Utx* zygotic expression.

### Robust regulatory networks allow proper Hox gene expression that masks fine regulation mediated by TnaA

A central contribution of the present work is that wild-type domains of Hox gene expression are not visibly altered in *tna* mutant larval imaginal discs, in spite of the adult mutant Hox phenotypes presented by these animals. These results make us consider that first, robust regulatory networks protect proper Hox gene expression and that the role of fine modulators such as TnaA is difficult to analyze in this scenario. Second, *tna* might be required in particular stages of development that we did not explore here, and third, that the *tna* mutant cells in imaginal discs that will produce the cuticular adult mutant phenotypes, might be reading very subtle differences in the Hox protein levels that we could not detect by immunostaining.

An argument to explain why the effect of *tna* mutations can only be observed in ectopic but not on wild-type regions of Hox expression derives from the robustness of regulatory networks. For example, *Ubx* has several enhancers (and at the end, all Hox genes) that ensure proper *Ubx* expression in time and space. Some of them are active in haltere discs in redundant spatial patterns which allows to buffer changes in *Ubx* expression levels due to natural variation [36]. Then it is possible that TnaA modulates the expression of only some components of these regulatory networks in imaginal discs, and when those components fail to function, the other ones "compensate" for Hox gene expression. This compensation mechanism has been observed in experiments studying the effect of loss-of-function alleles of TrxG genes. In these experiments, although the TrxG function is totally removed in mitotic clones, Hox expression (particularly *Ubx*) is partially restored in a "patchy" way, probably by these compensation mechanisms [34, 37]. Taking in account this situation it makes sense that it was in a *Pc*-defective background where we were able to observe the suppression by *tna* mutations of *Ubx* and *Scr* ectopic expression in imaginal discs.

That *tna* suppresses the extra-sex-combs adult cuticular phenotype in *Pc*-defective individuals caused by derepression of *Scr* is known [5], but this effect in imaginal discs was analyzed until this work. The suppression effect was observed in imaginal discs harboring the *tna*^*EY22029*^ allele that affects primarily the production of the TnaA_130_ isoform (this work), or in adult animals harboring the *tna*^*1*^ allele [5], that lack both TnaA_130_ and TnaA_123_ isoforms (this work). This is also similar to the effect of a null *brm* mutation in the suppression ectopic *Scr* expression caused by *Pc* mutants in imaginal discs [2].

To study the developmental window of *tna* requirement for Hox gene expression, we made clones at different stages of development, finding that *tna* may be required at early stages (3–4 h AEL). Animals with clones generated at this time did not survive. In contrast, clones generated later (24–48 h AEL) do survive, and adults present a reduced number of sex comb teeth similarly to the *Scr* loss-of-function phenotype presented by *tna* hypomorphic and knocked down mutants. This suggests that *tna* requirements may be biphasic as has been shown for other TrxG genes such as *Utx* (discussed in the previous section).

Individuals with mutations in RNA polymerase II and transcriptional factors that facilitate initiation [38], or elongation [39] present, as *tna* mutant individuals, Hox loss-of-function phenotypes. Of these, *kismet* (*kis*) is a TrxG gene involved in elongation that was identified because it suppresses ectopic expression of *Scr* in *Pc* heterozygotes [40]. *kis* clones induced during larval development do not show homeotic transformations, meanwhile clones induced earlier at the cellular blastoderm stage, show a reduction on sex comb teeth [41]. Many evidences points towards the possibility that TnaA could be required as a co-factor of the BRAHMA BAP chromatin remodeling complex, but it will also be possible that it targets other components of the general transcriptional machinery.

### TnaA on gene expression mediated by the BAP complex, and other general transcription factor targets

The BRAHMA BAP complex is required for the expression of multiple genes at different times of development [42]. TnaA physically interacts with the E2 Ubc9 SUMO-conjugating enzyme and with the subunits of the BRAHMA BAP complex, Osa and Brm [6]. TnaA could be modifying the assembly, the recruitment, or the remodeling function of the complex by stimulating the SUMOylation of one or more BAP subunits to facilitate Hox gene expression at a specific time, or cell-type. In fact, we have shown that TnaA co-localizes sometimes with the BAP subunit Osa in polytene chromosome bands of third instar larvae, but not in others [6], meaning that TnaA is required for function of the BAP complex at some gene targets but not in others, or that the co-localization of TnaA with Osa is transient. On the other side, TnaA may act on targets other than the BRAHMA BAP complex, meaning that epistatic relationships at different levels can contribute to the phenotypes derived from TnaA function(s) (S4 Fig).

Although TnaA itself is not a subunit of the BAP complex, *tna* defective clones in imaginal discs, resemble the behavior of defective clones in some subunits of the BAP complex. We compared our results particularly with the ones obtained with *brm* and *osa* clones, because TnaA physically and genetically interacts with the BAP subunits Brahma and Osa [5, 6]. Kassis *et al*., (2017) have recently published an excellent summary of results involving the clonal analyses of TrxG genes, and we only note here that germ line and/or somatic clones for mutant *brm*, *osa*, *Snf5-related 1* (*Snr1*), and *moira* (*mor*) [3, 4, 31, 43, 44], all of them encoding BAP subunits, do not present the same phenotypes, and show requirements at different times of development. For example, analyses of *brm* clones suggest defects in cell division and in adult peripheral nervous system [43]. *osa* clones in the germ line produce embryos with segmentation defects [4], and somatic clones in the wing imaginal discs, have defects in venation, and in cell growth and viability [31]. These clones do not present homeotic transformations although adult individuals with hypomorphic *brm* or *osa* mutations do have homeotic phenotypes, e. g. [40]. All these evidences, indicates that the BAP complex acts on different gene targets influenced by other factors, including TnaA.

In conclusion, these and other differences and mechanisms, may account for the diverse developmental requirements observed in the clonal analyses of different TrxG genes. As other TrxG genes which have functions in the regulation of genes other than the Hox, *tna* could have targets not related to Hox expression that are essential for larval or pupal survival. *tna* epistatic relationships, may involve different TnaA isoforms that could be required for the expression of different gene targets or at diverse times of development (S4 Fig).

If TnaA is influencing BRAHMA BAP complex function, it may act close to the promoter or on regulatory elements such as enhancers where BAP complexes are remodeling chromatin. TnaA may also have other targets than the BAP complex and have a wider target specificity as has been shown for other E3 SUMO ligases (reviewed in [8]). These are questions still unanswered and for example, chromatin immunoprecipitation experiments with TnaA antibodies that recognize specific isoforms at different times of development, will be helpful to start to determine the range of action of these TrxG proteins.

## Supporting information

S1 Fig Cell survival is not affected in TnaA defective mitotic clones. (**A**) *tna*^*1*^*/tna*^*1*^ (GFP^-/-^), and *tna*^*+*^*/tna*^*+*^ (GFP^+/+^) clones in a wing disc showing an example of the areas affected by clone-induction. (**B**) Comparison of the area of 13 *tna*^*1*^*/tna*^*1*^ (GFP^-/-^), and *tna*^*+*^*/tna*^*+*^ (GFP^+/+^) adjacent clones from independent events of clone-induction in wing discs. There were no significant (NS) differences between correspondant areas (t-test, P>0.05).
(TIF)

S2 Fig TnaA level does not affect Scr immunostaining in leg imaginal discs. TnaA (red) and Scr (green) immunostaining (red) of *tna*^*1*^*/+* or *tna*^*EY22029*^*/+* (upper panel), or *tna*^*1*^*/tna*^*EY22029*^ (lower panel) leg discs. DNA is stained with Hoescht (left) and images with merged TnaA and Scr signals is shown (extreme right). Note that TnaA level diminishes in *tna*^*1*^*/tna*^*EY22029*^ leg discs, although the Scr signal looks normal, and 77% of adult *tna*^*1*^*/tna*^*EY22029*^ animals present a loss-of-function Scr phenotype (Table 2).
(TIF)

S3 Fig Osa defective mitotic clones in haltere discs. *osa*^*308*^ mitotic clones were induced with the Ubx-FLPase. Immunostaining of Osa with the anti-Osa15A8 (dil. 1:200) in a haltere disc where mitotic clones were induced. DNA was stained with Hoechst (blue) to show nuclear presence. RFP (red) marks the *osa*^*+*^/*osa*^*-*^ cells that did not recombine (middle red intensity), and the *osa*^*+*^/*osa*^*+*^ cells result of the recombination event (strong red intensity). RFP^-^ marks the *osa*^*308*^/*osa*^*308*^ clone, as corroborated by the absence of Osa immunostaining (green).
(TIF)

S4 Fig Possible TnaA targets that can influence gene expression involved in organism survival and Hox loss-of-function phenotypic outcomes. Representation of TnaA target proteins that can influence the transcription of different genes. Epistatic relationships, can contribute to the Hox loss-of-function and organism survival phenotypes studied in this work.
(TIF)
