# Supplementary figures and images for: The role of the trithorax group TnaA isoforms in Hox gene expression, and in Drosophila late development
Source: PLoS One. 2018 Oct 29;13(10):e0206587. doi: 10.1371/journal.pone.0206587 (PMC6205608; doi:10.1371/journal.pone.0206587)

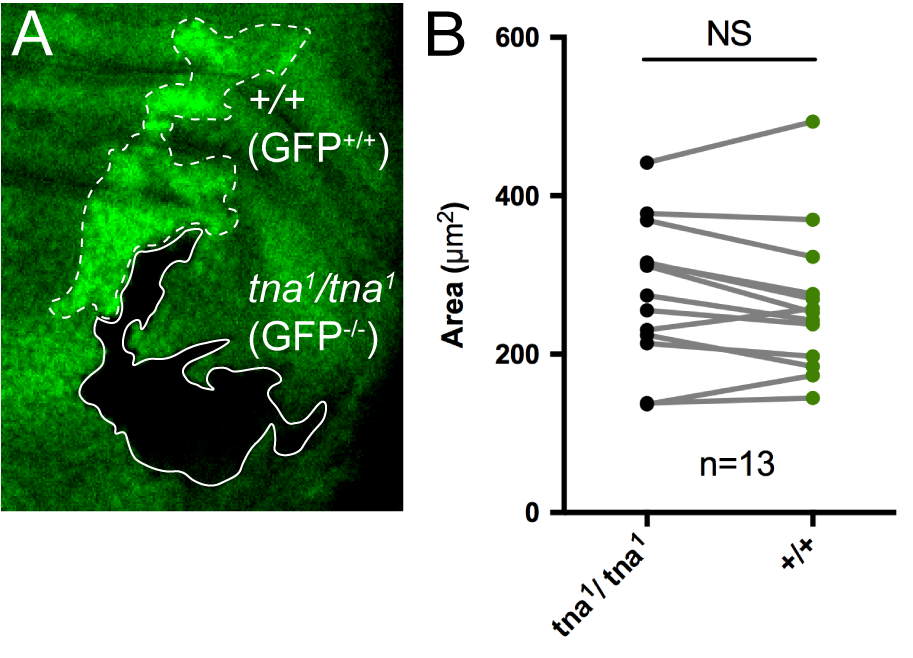

Supplement: S1 Fig — (A) tna1/tna1 (GFP-/-), and tna+/tna+ (GFP+/+) clones in a wing disc showing an example of the areas affected by clone-induction. (B) Comparison of the area of 13 tna1/tna1 (GFP-/-), and tna+/tna+ (GFP+/+) adjacent clones from independent events of clone-induction in wing discs. There were no significant (NS) differences between correspondant areas (t-test, P>0.05). (TIF) [file pone.0206587.s001.tif]

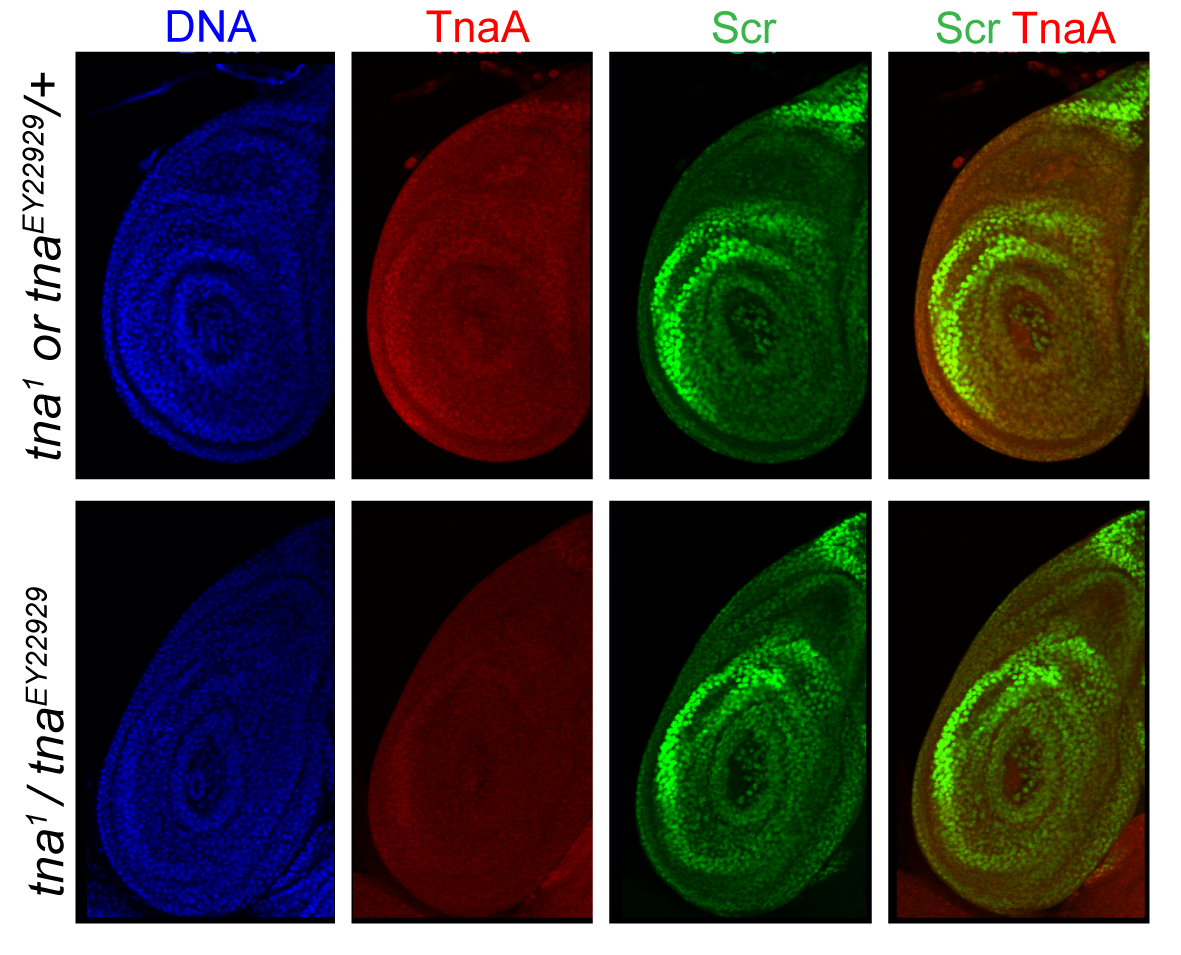

Supplement: S2 Fig — TnaA (red) and Scr (green) immunostaining (red) of tna1/+ or tnaEY22029/+ (upper panel), or tna1/tnaEY22029 (lower panel) leg discs. DNA is stained with Hoescht (left) and images with merged TnaA and Scr signals is shown (extreme right). Note that TnaA level diminishes in tna1/tnaEY22029 leg discs, although the Scr signal looks normal, and 77% of adult tna1/tnaEY22029 animals present a loss-of-function Scr phenotype (Table 2). (TIF) [file pone.0206587.s002.tif]

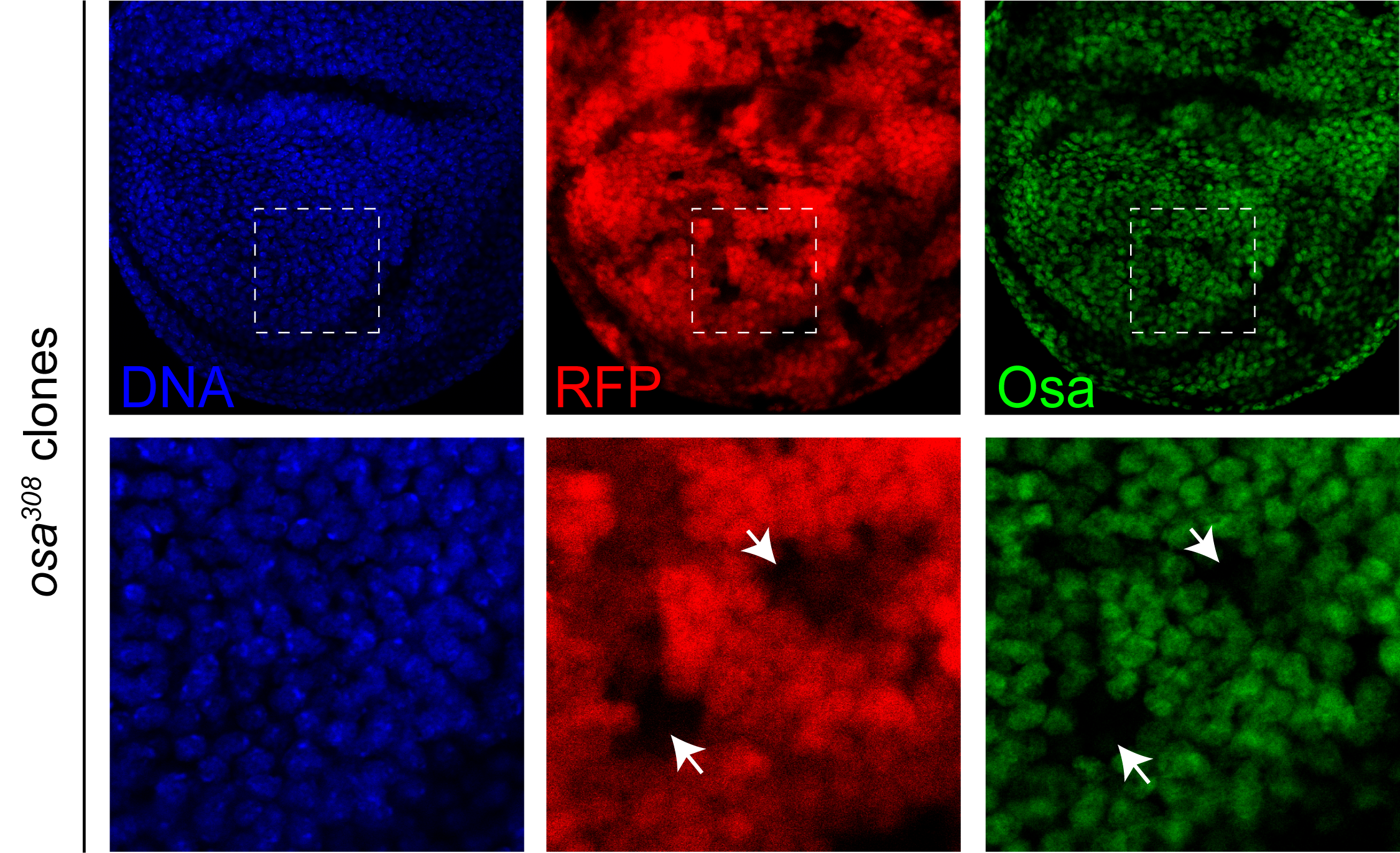

Supplement: S3 Fig — osa308 mitotic clones were induced with the Ubx-FLPase. Immunostaining of Osa with the anti-Osa15A8 (dil. 1:200) in a haltere disc where mitotic clones were induced. DNA was stained with Hoechst (blue) to show nuclear presence. RFP (red) marks the osa+/osa- cells that did not recombine (middle red intensity), and the osa+/osa+ cells result of the recombination event (strong red intensity). RFP- marks the osa308/osa308 clone, as corroborated by the absence of Osa immunostaining (green). (TIF) [file pone.0206587.s003.tif]

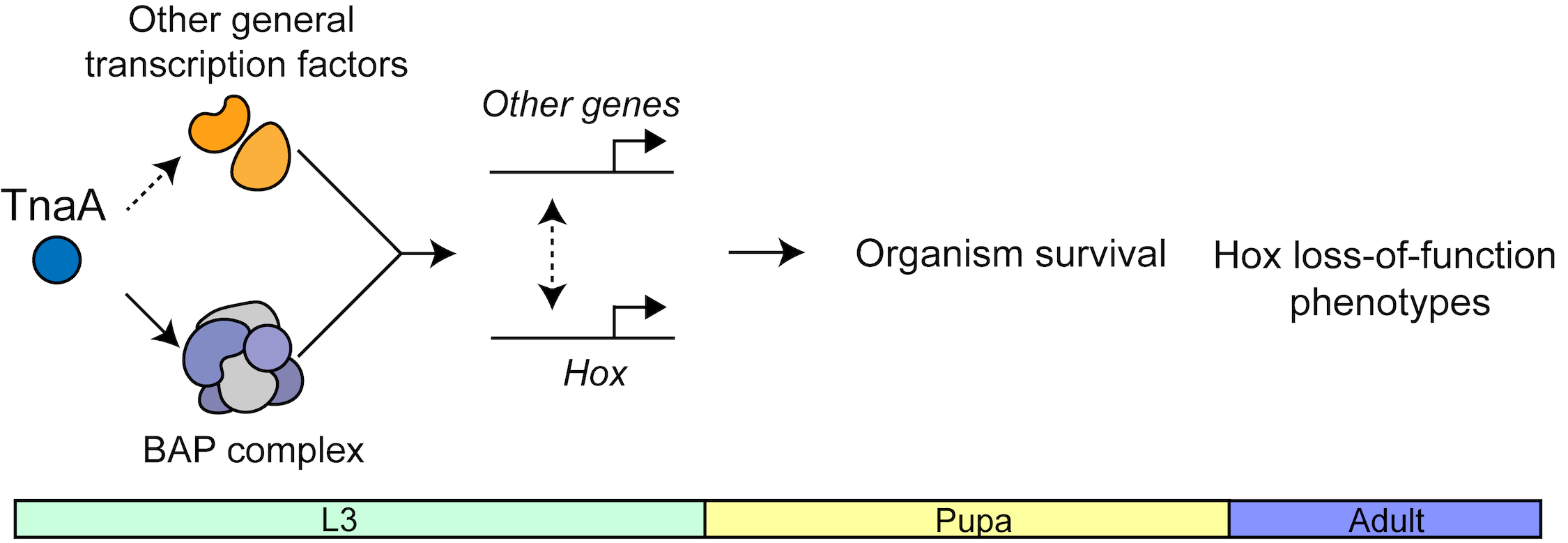

Supplement: S4 Fig — Representation of TnaA target proteins that can influence the transcription of different genes. Epistatic relationships, can contribute to the Hox loss-of-function and organism survival phenotypes studied in this work. (TIF) [file pone.0206587.s004.tif]
